# Integrating epidemiologic modeling and explainable machine learning to evaluate body roundness index for WHO-defined high cardiovascular risk: evidence from the ChinaHEART-Luohe screening cohort

**DOI:** 10.3389/fnut.2026.1818427

**Published:** 2026-04-21

**Authors:** Zhiwei Huang, Jirui Cai, Yang Liu, Jin Wang, Yabo Huang, Junxiang Liu, Li Wu, Haiqin Yuan, Jing Bai, Guirang Zhao, Qiaotao Xie, Haoran Wang

**Affiliations:** 1 Henan University College of Medicine, Henan University, Kaifeng, China; 2 Luohe Central Hospital, Luohe Medical College, Luohe, China; 3 Huaihe Hospital of Henan University, Kaifeng, China; 4 Huanghua People's Hospital, Cangzhou, China; 5 Luohe Center for Disease Control and Prevention, Luohe, China; 6 Henan Key Laboratory of Fertility Protection and Aristogenesis, Luohe, China

**Keywords:** body roundness index, cardiovascular risk, community screening, logistic regression, machine learning, obesity phenotype, SHAP, WHO CVD risk charts

## Abstract

**Background:**

Efficient identification of individuals at high cardiovascular disease (CVD) risk is essential for prevention in middle-aged and older adults. The body roundness index (BRI), derived from waist circumference and height, may capture body-shape-related risk beyond conventional measures. We examined the association of BRI with World Health Organization (WHO)-defined CVD high-risk status in a community-based screening population.

**Methods:**

This cross-sectional study used baseline data from the Luohe branch of the ChinaHEART cohort, a community-based health screening program in Luohe, Henan, China (March 2021 to February 2022), including adults aged 35–75 years. WHO-defined CVD high-risk status was determined using WHO CVD risk charts, with an estimated 10-year risk ≥20% classified as high risk. BRI was analyzed as a continuous variable (per 1-unit increase), quartiles, and a binary variable using a receiver operating characteristic (ROC)-derived threshold. Multivariable logistic regression, restricted cubic splines, ROC analysis with bootstrap confidence intervals, and subgroup/interaction analyses were performed. An explainable machine-learning workflow (LASSO, random forest, and SHAP) was also applied.

**Results:**

Among 6,858 participants, 1,489 (22%) were classified as WHO-defined CVD high risk. Higher BRI remained associated with high-risk status in fully adjusted models. ROC analysis showed only modest standalone discrimination, while subgroup analyses suggested heterogeneity by sex and cardiometabolic strata. In machine-learning analyses, BRI was retained among selected predictors and contributed meaningfully within the multivariable model.

**Conclusion:**

In this community screening population, BRI was positively associated with WHO-defined CVD high-risk status and may serve as a low-cost adjunct marker to prioritize individuals for comprehensive risk evaluation in primary-care screening settings.

## Introduction

Cardiovascular disease (CVD) remains a leading cause of morbidity and mortality worldwide (1–3). In many settings, particularly in primary-care and community screening programs, the ability to rapidly identify individuals at high risk is central to prevention strategies. Risk stratification tools based on demographic and clinical predictors can support decision-making; however, the performance, feasibility, and acceptability of risk assessment approaches vary across populations and resource contexts.

Obesity and adverse fat distribution are widely recognized contributors to cardiometabolic risk (4–10). While body mass index (BMI) is commonly used, it does not capture body shape or central adiposity patterns that may better reflect metabolic dysfunction. Waist circumference provides additional information, but single anthropometric measures may still fail to reflect the integrated geometry of the human body associated with cardiometabolic risk. Derived indices that combine anthropometry into interpretable constructs may improve practicality and risk communication in screening.

The body roundness index (BRI) is an anthropometric index calculated from waist circumference and height, intended to approximate body shape and fat distribution (8, 11–17). BRI is attractive because it can be computed from routine measurements without specialized equipment. If BRI is associated with validated CVD risk classification, it could serve as a pragmatic adjunct marker to prioritize individuals for more intensive evaluation or prevention efforts, especially in large-scale community screening where time and resources are limited.

The World Health Organization (WHO) CVD risk charts have been used internationally to estimate the 10-year risk of a major cardiovascular event and to categorize individuals into clinically meaningful risk groups (18, 19). In large-scale screening programs, a standardized, algorithm-based risk classification provides a consistent outcome definition suitable for studying the association between anthropometric indices and risk status.

Using baseline data from a branch of the ChinaHEART cohort, a large community screening program conducted in Luohe, China, we investigated whether BRI is associated with WHO-defined CVD high-risk status (20, 21). We assessed (i) the association between BRI and high-risk status using multivariable logistic regression, (ii) potential non-linear dose–response relationships using restricted cubic splines, (iii) discriminatory performance and an operational BRI threshold using receiver operating characteristic (ROC) analyses with bootstrap-based confidence intervals, (iv) heterogeneity using subgroup and interaction analyses, and (v) the role of BRI within an explainable machine-learning framework (least absolute shrinkage and selection operator [LASSO] selection, random forest [RF] modeling, shapely additive explanations [SHAP] interpretation).

## Methods

### Study design, setting, and population

This cross-sectional study was conducted in accordance with the Strengthening the Reporting of Observational Studies in Epidemiology (STROBE) statement and the Declaration of Helsinki (22, 23). It was based on a branch of the ChinaHEART cohort, a government-supported public health initiative aimed at identifying CVD risk and implementing targeted community interventions nationwide (20, 21). Community residents were recruited from township hospitals in Luohe, Henan Province, China, between March 2021 and February 2022. Eligible participants were aged 35–75 years, were permanent residents of the project site (≥6 months residence in the preceding 12 months), and were willing and able to complete questionnaire interviews and undergo clinical examinations; written informed consent was obtained from all participants. Individuals with acute cardiovascular events, end-stage organ failure, malignant tumors, severe trauma, or cognitive impairment that precluded questionnaire completion were excluded. A total of 6,860 participants were enrolled; for the present analysis, participants with missing data required to compute BRI, determine WHO CVD risk classification, or extract prespecified covariates were excluded, leaving 6,858 participants for final analyses. The study protocol was approved by the institutional ethics committee of Fuwai Hospital and filed with the local ethics committee of Luohe Central Hospital.

### Data collection and measurement procedures

Trained healthcare staff collected data using standardized procedures and a unified measurement protocol to ensure consistency across screening sites. All interview-based variables were obtained via face-to-face interviews conducted by trained staff using standardized questionnaires. When applicable, medication use was recorded based on participant report and/or available medication records.

Demographic variables included age (recorded in years), sex (recorded as male or female), ethnicity (recorded according to the program’s coding scheme), education (for analysis, recorded/categorized as illiteracy status: illiterate [yes/no] according to the program’s coding scheme), and marital status (recorded as married [yes/no]).

Lifestyle behaviors included smoking status and alcohol consumption. Smoking status was recorded as current smoking (yes/no). For risk chart computation, “current smoking” included individuals who currently smoked and those who had stopped smoking within the past year, aligning with the risk stratification algorithm used in the screening setting. Alcohol consumption was recorded as current alcohol use (yes/no) according to the program’s definition (self-reported regular drinking in the recent period).

Medical history variables included hypertension, diabetes mellitus, and stroke. Hypertension was recorded as a history of hypertension (yes/no), based on participant report of prior diagnosis and/or ongoing antihypertensive therapy. Diabetes mellitus was recorded as a history of diabetes (yes/no); for risk chart computation, diabetes was defined based on prior diagnosis and/or use of glucose-lowering medications or insulin. Stroke was recorded as a history of stroke (yes/no), based on participant report of a prior stroke diagnosis; when available, we further sought medical documentation to validate self-reported results.

Medication use was recorded as binary indicators (yes/no) for the following classes: antihypertensive drug therapy, antidiabetic drug therapy, statin use, and antiplatelet use.

Anthropometric measurements were performed according to a unified protocol by trained staff using calibrated equipment. Height was measured to the nearest 0.1 cm with participants standing without shoes. Weight was measured to the nearest 0.1 kg with participants in light clothing. Waist circumference was measured at the midpoint between the lower margin of the last rib and the top of the iliac crest, at the end of a gentle expiration, using a non-stretchable tape.

Blood pressure was measured using validated electronic sphygmomanometers in the seated position after at least 5 min of rest. Two measurements were taken and averaged. Heart rate was recorded during the measurement session (as provided by the device or recorded simultaneously) and used as beats per minute (bpm). Accordingly, systolic blood pressure (SBP) and diastolic blood pressure (DBP) were defined as the averages of two readings (mmHg), and heart rate was recorded in bpm.

Laboratory measurements were obtained from fasting blood samples collected after an overnight fast. Blood specimens were processed using standardized and regularly calibrated devices in accordance with quality-control procedures. The biomarkers used in the present analysis included triglycerides (TGs), total cholesterol (TC), high-density lipoprotein cholesterol (HDL-C), low-density lipoprotein cholesterol (LDL-C), non-high-density lipoprotein cholesterol (non-HDL-C), and fasting plasma glucose (fasting glucose).

### Exposure definition: body roundness index (BRI)

BRI was calculated using waist circumference and height (11). The index is derived from a geometrical model and is commonly expressed as: BRI = 364.2–365.5 × √{1 − [(WC/(2π)) (2)/(0.5 × Height) (2)]}.

Here, WC is waist circumference (meters), and Height is height (meters). BRI was analyzed in three ways: (1) Continuous: per 1-unit increase in BRI. (2) Quartiles: Q1 (lowest) through Q4 (highest), using Q1 as the reference. (3) Binary group: high vs. low BRI based on an ROC-derived threshold.

### Outcome definition: WHO-defined CVD high-risk status

CVD high-risk status was defined using the WHO CVD risk charts (18, 19). The screening system estimated each participant’s 10-year CVD risk based on inputs required by the chart algorithm (including age, sex, averaged SBP, smoking status, diabetes status, and total cholesterol as implemented in the screening workflow). Participants were classified as “high risk” if the estimated 10-year risk was ≥20% and “non-high risk” if it was <20%.

### Covariates and model specification

Covariates were selected *a priori* based on clinical relevance and feasibility in the screening context. We adopted a stepwise adjustment approach to evaluate robustness while avoiding overadjustment for variables that are direct components of the WHO risk chart. Model 1 (crude): BRI only. Model 2: adjusted for alcohol consumption and triglycerides. Model 3: additionally adjusted for education, marital status (married), diastolic blood pressure, and heart rate. These variables were selected to reflect routinely collected non-chart socioeconomic and hemodynamic factors in the screening setting, while limiting circular adjustment for variables already embedded in the WHO risk-chart outcome.

Because the WHO risk chart already incorporates major determinants such as age, sex, SBP, smoking, diabetes, and cholesterol, we did not include the full set of chart components as covariates in the association models to reduce circularity and collinearity. Instead, we focused on non-chart covariates that may confound the relationship between body shape and risk status and that were consistently collected in the screening setting.

### Statistical analyses

All analyses were performed using R (version 4.5.2). A two-sided *p*-value <0.05 was considered statistically significant. Participants were summarized overall and by BRI group. Continuous variables were presented as median (Q1, Q3) and compared using the Mann–Whitney U test. Categorical variables were presented as n (%) and compared using Pearson’s chi-square test. We used logistic regression to estimate odds ratios (ORs) and 95% confidence intervals (CIs) for the association of BRI with CVD high-risk status. BRI was evaluated as continuous (per 1-unit increase), quartiles, and a binary group. Results were reported for Models 1–3 as specified above. To explore potential non-linear dose–response relationships, we fitted restricted cubic spline (RCS) functions for continuous BRI in logistic regression models. We tested for non-linearity using likelihood ratio tests comparing the spline model to a linear term model. Predicted probabilities and confidence bands were visualized. We evaluated discrimination of BRI and multivariable models using ROC curves and area under the curve (AUC). To quantify uncertainty, we calculated bootstrap-based confidence intervals for AUC and for the Youden index-derived optimal cutoff of BRI. The “optimal” cutoff was used to define an empirical threshold, and we reported a pragmatic operational cutoff (rounded) for grouping. We conducted subgroup analyses to examine heterogeneity in the BRI-risk association across strata of age (quartiles), sex, education, marital status, alcohol consumption, DBP (quartiles), heart rate (quartiles), and triglycerides (quartiles). Interaction was assessed by adding multiplicative interaction terms and reporting *p*-values for interaction. Because multiple subgroup comparisons were performed, we additionally controlled the false discovery rate using the Benjamini–Hochberg procedure for subgroup-specific association *p*-values and interaction *p*-values. Subgroup analyses were conducted for both continuous BRI (per 1-unit increase) and binary BRI group (ROC-derived cutoff). Because multiple subgroup and interaction analyses were performed, these analyses should be considered exploratory and hypothesis-generating. We therefore interpreted subgroup-specific findings cautiously, with particular emphasis on the overall pattern, effect sizes, and confidence intervals rather than on nominal p-values alone.

Additionally, supplementary analyses were performed to compare BRI with body mass index (BMI) and waist circumference (WC) in anthropometric-only models and after sequential addition to the Model 2 and Model 3 base covariate sets. Incremental discrimination was assessed by changes in AUC (ΔAUC) using DeLong tests for paired ROC curves and by continuous net reclassification improvement (NRI), with 95% confidence intervals estimated from 1,000 bootstrap resamples. We further performed decision-curve analysis across threshold probabilities of 0.01–0.50 and a sensitivity analysis using a reduced Model 3 base set excluding triglycerides and diastolic blood pressure before re-adding BRI.

### Explainable machine-learning analyses (LASSO + RF + SHAP)

To explore the relative importance of BRI within a multivariable context, we implemented an explainable machine-learning workflow. Candidate predictors were prespecified based on variables available and consistently collected in the screening setting, including BRI and selected demographic and cardiometabolic variables. We first applied 10-fold cross-validated LASSO logistic regression using AUC as the optimization metric and selected predictors at the 1-standard-error rule (λ1se). Predictors with non-zero coefficients were then carried forward to random forest modeling, while BRI was retained in the candidate set for interpretation.

For random forest analysis, the data were randomly partitioned into a training set (70%) and a test set (30%). The model was trained with 500 trees, and the number of variables randomly sampled at each split was set to the square root of the number of input features (rounded down to the nearest integer, with a minimum of 1). No additional class-rebalancing procedure was applied. Model performance was evaluated in the held-out test set using the AUC.

SHAP (Shapley Additive Explanations) values were calculated using the fastshap framework with Monte Carlo simulation (30 repetitions) based on the trained random forest model. SHAP summary plots were used to display both the relative importance of each predictor and the direction of its contribution to the modeled probability of WHO-defined high-risk status.

## Results

### Participant characteristics

**Figure 1:**
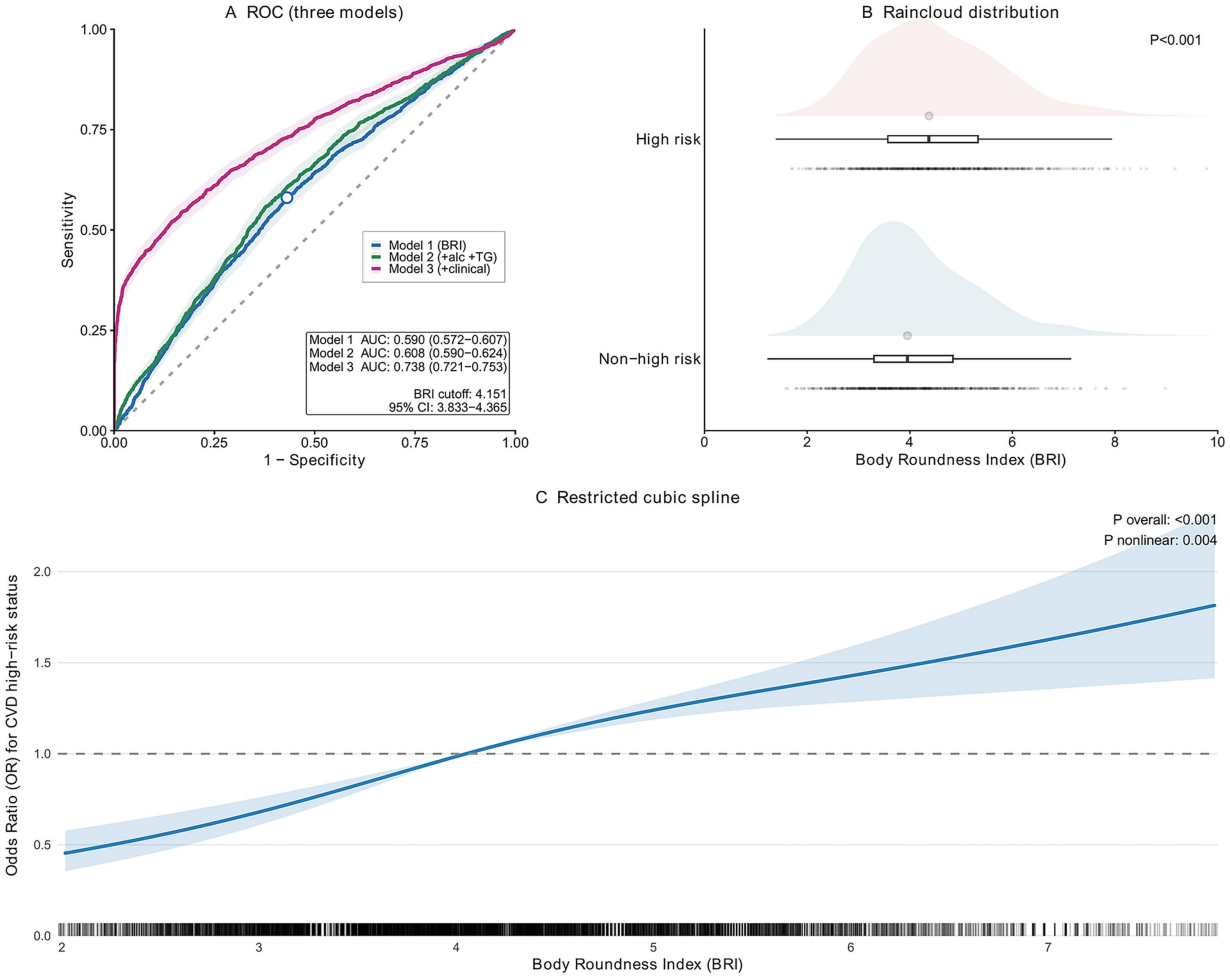
Association between body roundness index and WHO-defined cardiovascular disease high-risk status. **(A)** Receiver operating characteristic (ROC) curves for models classifying WHO-defined cardiovascular disease (CVD) high-risk status, including a BRI-only model and models with additional routinely collected covariates. **(B)** Raincloud plot showing the distribution of body roundness index (BRI) by CVD high-risk status. **(C)** Restricted cubic spline (RCS) depicting the dose–response relationship between continuous BRI and the odds of CVD high-risk status, with 95% confidence interval.

A total of 6,858 participants were included in the analysis. The median age was 58 years (Q1–Q3: 51–66), and 2,586 (38%) were men. Overall, 1,489 participants (22%) met criteria for WHO-defined CVD high-risk status (Table 1). Consistent with the raincloud distribution in Figure 1B, BRI values were significantly higher in participants classified as CVD high risk than in those classified as non-high risk (*p* < 0.001).

**Table 1:** Baseline characteristics by BRI group (cutoff = 4).

Variable	Overall *N* = 6,858	Low BRI (<4) *N* = 3,312	High BRI (≥4) *N* = 3,546	*p*-value
Age (years)	58 (51, 66)	57 (48, 66)	59 (53, 67)	<0.001
Sex				<0.001
Male	2,586 (38%)	1,333 (40%)	1,253 (35%)	
Female	4,272 (62%)	1,979 (60%)	2,293 (65%)	
Ethnicity				0.006
Han Chinese	6,772.0 (98.7%)	3,281.0 (99.1%)	3,491.0 (98.4%)	
Mongolian	3.0 (0.0%)	3.0 (0.1%)	0.0 (0.0%)	
Hui	83.0 (1.2%)	28.0 (0.8%)	55.0 (1.6%)	
Education	5,566 (81%)	2,819 (85%)	2,747 (77%)	<0.001
Married	6,087 (89%)	2,986 (90%)	3,101 (87%)	<0.001
Current smoking	1,371 (20%)	708 (21%)	663 (19%)	0.006
Alcohol consumption	380 (5.5%)	147 (4.4%)	233 (6.6%)	<0.001
Height (cm)	159 (154, 166)	161 (155, 168)	158 (153, 164)	<0.001
Weight (kg)	64 (58, 72)	60 (54, 66)	68 (62, 75)	<0.001
BMI (kg/m^2^)	25 (23, 27)	23 (22, 25)	27 (25, 29)	<0.001
Waist circumference (cm)	86 (80, 92)	80 (76, 84)	92 (87, 97)	<0.001
Systolic blood pressure (mmHg)	137 (126, 150)	133 (123, 146)	143 (131, 152)	<0.001
Diastolic blood pressure (mmHg)	84 (77, 91)	81 (75, 88)	86 (78, 94)	<0.001
Heart rate (bpm)	76 (70, 83)	75 (70, 82)	76 (70, 83)	0.082
Triglycerides (mmol/L)	1.51 (1.14, 2.03)	1.41 (1.07, 1.75)	1.66 (1.24, 2.29)	<0.001
HDL-C (mmol/L)	1.43 (1.23, 1.66)	1.46 (1.26, 1.70)	1.38 (1.19, 1.61)	<0.001
LDL-C (mmol/L)	2.63 (2.07, 3.15)	2.63 (2.10, 3.05)	2.62 (2.04, 3.24)	0.123
Non-HDL-C (mmol/L)	3.31 (2.66, 3.98)	3.19 (2.55, 3.79)	3.44 (2.78, 4.16)	<0.001
Fasting glucose (mmol/L)	5.40 (5.20, 6.00)	5.30 (5.10, 5.80)	5.60 (5.30, 6.30)	<0.001
Hypertension	1,578 (23%)	445 (13%)	1,133 (32%)	<0.001
Diabetes mellitus	428 (6.2%)	129 (3.9%)	299 (8.4%)	<0.001
Stroke	192 (2.8%)	67 (2.0%)	125 (3.5%)	<0.001
CVD high-risk status	1,489 (22%)	550 (17%)	939 (26%)	<0.001
Family history of stroke	157 (2.3%)	60 (1.8%)	97 (2.7%)	0.011
Antidiabetic drug therapy	266 (3.9%)	76 (2.3%)	190 (5.4%)	<0.001
Antihypertensive drug therapy	915 (13%)	240 (7.2%)	675 (19%)	<0.001
Statin use	207 (3.0%)	54 (1.6%)	153 (4.3%)	<0.001
Antiplatelet use	104 (1.5%)	31 (0.9%)	73 (2.1%)	<0.001

Continuous variables: median (Q1, Q3). Binary variables are shown as n (%) for value = 1 only. Sex is displayed as categorical (all levels). Youden cutoff (real) for BRI was 4.151, but the grouping cutoff used here is 4. Mann–Whitney U test; Chi-Square test.

Using the operational cutoff (BRI ≥ 4 vs. <4), participants with high BRI were generally older and showed less favorable anthropometric, blood-pressure, and metabolic profiles than those with low BRI. WHO-defined CVD high-risk status was also more common in the high-BRI group (Table 1).

### Association between BRI and WHO-defined CVD high-risk status

In logistic regression, treating BRI as a continuous exposure, each 1-unit increase in BRI was associated with higher odds of CVD high-risk status. In the crude model, the OR per 1-unit increase was 1.252. After adjustment for alcohol consumption and triglycerides (Model 2), the association remained similar (OR 1.218). In the fully adjusted model (Model 3), the association persisted though attenuated (OR 1.078). When analyzed in quartiles, participants in the highest quartile (Q4) had markedly higher odds of high-risk status compared with Q1 in crude analyses. After full adjustment, the Q4 vs. Q1 association remained statistically significant. Intermediate quartiles showed smaller or non-significant associations after adjustment. Using the operational binary group (BRI ≥ 4 vs. <4), high BRI was associated with greater odds of CVD high-risk status across models. The crude OR was 1.791; after full adjustment, the OR remained significant albeit attenuated (Table 2).

**Table 2:** Associations of BRI with CVD high-risk status (three models).

Exposure	Model 1		Model 2		Model 3	
	OR (95% CI)	*p*-value	OR (95% CI)	*p*-value	OR (95% CI)	*p*-value
BRI (per 1-unit increase)	1.252 (1.198, 1.309)	<0.001	1.218 (1.163, 1.276)	<0.001	1.078 (1.025, 1.134)	0.004
BRI group (high vs. low; cutoff = 4)	1.791 (1.590, 2.016)	<0.001	1.671 (1.481, 1.887)	<0.001	1.247 (1.093, 1.423)	0.001
BRI quartiles (Q2 vs. Q1)	1.112 (0.928, 1.332)	0.250	1.061 (0.885, 1.272)	0.523	0.957 (0.788, 1.161)	0.653
BRI quartiles (Q3 vs. Q1)	1.665 (1.403, 1.976)	<0.001	1.553 (1.306, 1.846)	<0.001	1.183 (0.982, 1.426)	0.077
BRI quartiles (Q4 vs. Q1)	2.173 (1.839, 2.567)	<0.001	1.950 (1.644, 2.314)	<0.001	1.291 (1.072, 1.555)	0.007

Model 1: crude (BRI only). Model 2: adjusted for alcohol consumption and triglycerides. Model 3: additionally adjusted for Married, education, diastolic blood pressure, and heart rate. BRI binary grouping uses a cutoff = 4 (high: BRI ≥ 4). Quartiles are based on the sample distribution (Q1 as reference).

### Dose–response pattern

Restricted cubic spline models demonstrated a significant positive and non-linear association between BRI and the odds of WHO-defined CVD high-risk status (*P* overall <0.001; *P* for non-linearity = 0.004) (Figure 1C). The curve suggested a relatively modest change at lower BRI values, followed by a steeper increase as BRI rose, indicating that the risk increment was not constant across the BRI distribution. This pattern was consistent with the multivariable logistic regression, in which higher BRI remained associated with increased odds of CVD high-risk status. Visually, the increase in odds appeared more pronounced as BRI approached approximately 4, after which the curve became steeper. This suggests that the adverse association between body roundness and a WHO-defined high-risk status may be stronger in the higher-BRI range rather than increase uniformly across the entire distribution.

### Discrimination performance and cutoff determination

In ROC analyses, BRI alone demonstrated modest discrimination for identifying WHO-defined CVD high-risk status (AUC 0.590, 95% CI 0.572–0.607). The AUC improved when additional covariates were incorporated (M2: AUC 0.608, 95% CI 0.590–0.624; M3: AUC 0.738, 95% CI 0.721–0.753). The Youden index suggested an optimal BRI cutoff of 4.151 with bootstrap confidence intervals, supporting the pragmatic use of BRI ≥ 4 as an operational threshold for grouping in descriptive and screening-oriented contexts (Figure 1A).

In supplementary analyses (Supplementary Figure S1), BRI showed anthropometric-only discrimination similar to WC and slightly better than BMI. Adding BRI, BMI, or WC to the Model 2 base model improved discrimination, with the largest numerical gain observed for BRI (ΔAUC 0.041, 95% CI 0.028–0.055; continuous NRI 0.260, 95% CI 0.203–0.317). By contrast, incremental gains over the Model 3 base model were limited: BRI showed only a modest improvement (ΔAUC 0.002, 95% CI 0.000–0.003; *p* = 0.010; NRI 0.104, 95% CI 0.044–0.156), whereas BMI showed no clear added value, and WC only a marginal improvement. Decision-curve analyses showed largely overlapping net-benefit curves across BRI, BMI, and WC, suggesting limited practical separation among these indices. In a sensitivity analysis using a reduced Model 3 base set excluding triglycerides and diastolic blood pressure, re-adding BRI still improved discrimination (ΔAUC 0.040, 95% CI 0.027–0.054; continuous NRI 0.266, 95% CI 0.211–0.322), indicating that its incremental value was unlikely to be fully explained by overlap with these variables. However, the sensitivity model showed lower net benefit than the full Model 3 + BRI model across most thresholds in Panel F.

### Subgroup and interaction analyses

**Figure 2:**
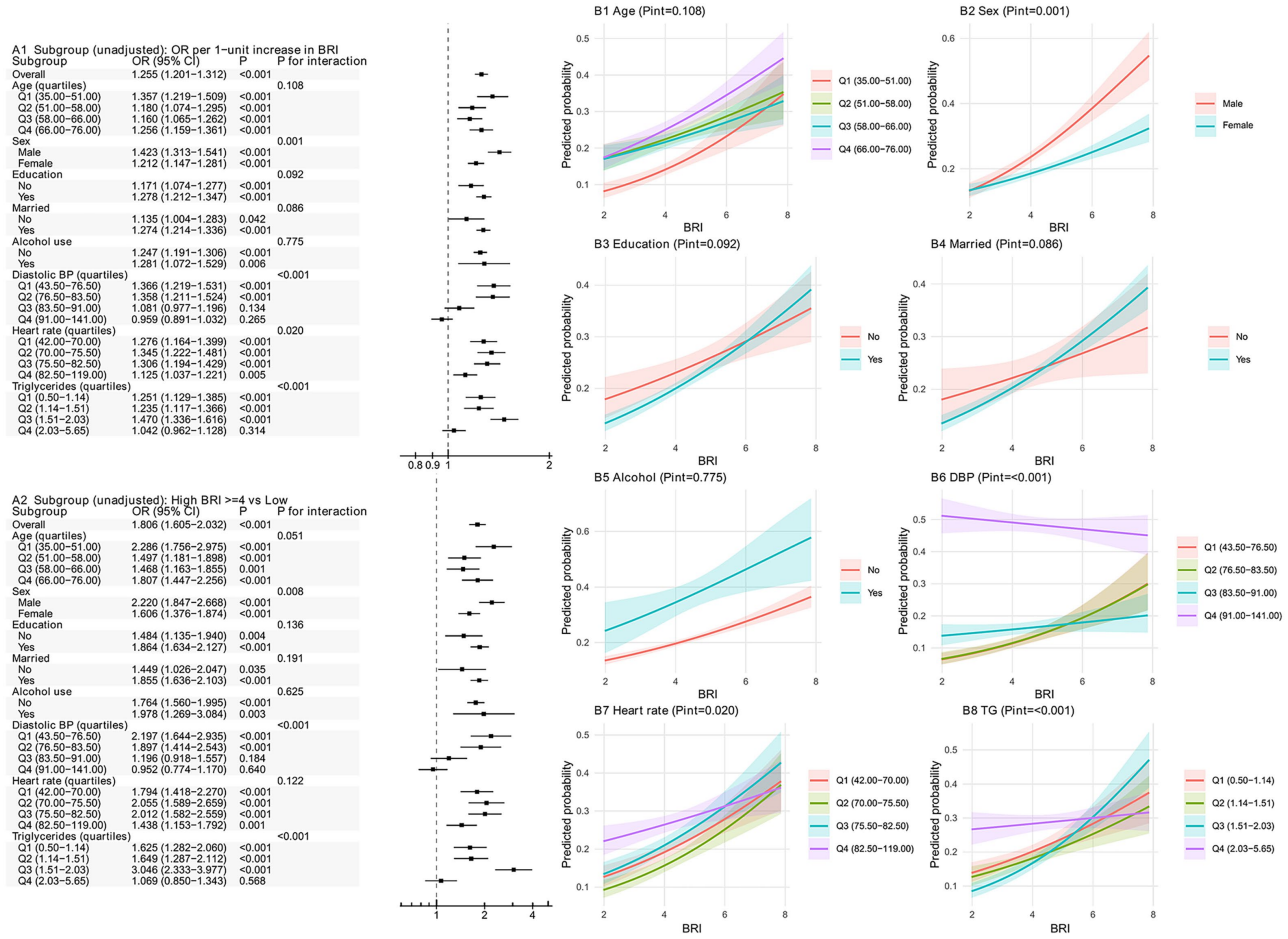
Subgroup and interaction analyses for body roundness index and WHO-defined cardiovascular disease high-risk status. **(A1)** Forest plot of unadjusted odds ratios (ORs) and 95% confidence intervals (CIs) per 1-unit increase in BRI across prespecified subgroups. **(A2)** Forest plot of unadjusted ORs and 95% CIs comparing high versus low BRI using an operational cutoff. **(B1–B8)** Interaction plots showing model-predicted probability of CVD high-risk status across the BRI range within subgroup strata (age quartiles, sex, education, marital status, alcohol use, diastolic blood pressure quartiles, heart rate quartiles, and triglyceride quartiles).

In unadjusted subgroup analyses, BRI was positively associated with WHO-defined CVD high-risk overall, whether modeled continuously (per 1-unit increase: OR 1.255, 95% CI 1.201–1.312; *p* < 0.001) or dichotomized using the ROC-derived cutoff approximated to BRI ≥ 4 (high vs. low: OR 1.806, 95% CI 1.605–2.032; *p* < 0.001) (Figures 2A1,A2). Subgroup analyses were conducted among participants with non-missing subgroup-defining variables; therefore, the ‘overall’ estimates in subgroup analyses may differ slightly from those in Table 2.

Effect modification was observed in several strata (Figures 2B1–B8). Sex showed significant heterogeneity in both parameterizations (*P* for interaction = 0.001 for continuous BRI; 0.008 for BRI ≥ 4 vs. <4), with larger effect estimates in men than women. DBP quartiles and TG quartiles also demonstrated significant interaction (both *P* for interaction <0.001 in the continuous and dichotomized analyses). Importantly, although estimates were generally directionally positive, the association was attenuated and not statistically significant in the highest DBP quartile (Q4) (per 1-unit BRI: OR 0.959, 95% CI 0.891–1.032; *p* = 0.265; BRI ≥ 4 vs. <4: OR 0.952, 95% CI 0.774–1.170; *p* = 0.640) and in the highest TG quartile (Q4) (per 1-unit BRI: OR 1.042, 95% CI 0.962–1.128; *p* = 0.314; BRI ≥ 4 vs. <4: OR 1.069, 95% CI 0.850–1.343; *p* = 0.568).

For heart rate, interaction was evident when BRI was analyzed continuously (*P* for interaction = 0.020) but not after dichotomization (*P* for interaction = 0.122), consistent with information loss/power reduction when collapsing a continuous exposure into a binary group. Age, education, marital status, and alcohol use did not show clear interaction in the continuous analysis (all *p* > 0.05), while age showed only borderline interaction in the dichotomized analysis (*p* = 0.051). The interaction plots of predicted probability were concordant with these findings, visually highlighting differences in the BRI-risk gradients across strata (notably by sex, DBP quartiles, and TG quartiles). After Benjamini–Hochberg FDR adjustment (Supplementary Table S1), the interaction signals for sex, DBP quartiles, and TG quartiles remained significant in both parameterizations, whereas the heart-rate interaction remained significant only in the continuous analysis, and the remaining subgroup interaction signals were not robust after multiplicity correction.

### Explainable machine-learning findings

**Figure 3:**
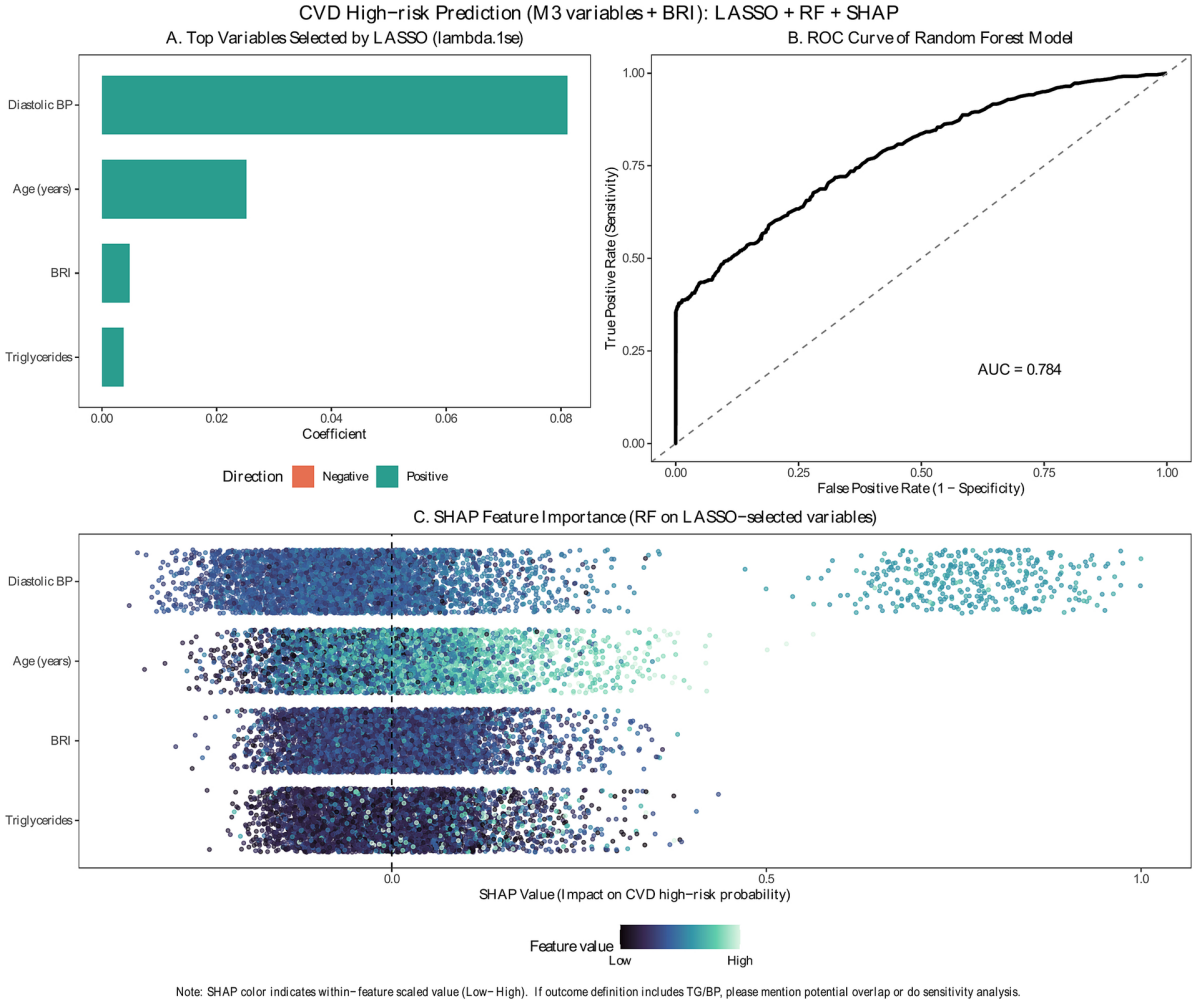
Explainable machine-learning workflow for modeling WHO-defined cardiovascular disease high-risk status. **(A)** Least absolute shrinkage and selection operator (LASSO) logistic regression with cross-validation for feature selection; predictors with non-zero coefficients at the selected penalty are shown. **(B)** Receiver operating characteristic (ROC) curve for the random forest (RF) model trained on LASSO-selected predictors. **(C)** Shapley additive explanations (SHAP) summary plot for the random forest model. Each point represents one participant; the horizontal position indicates the direction and magnitude of that feature’s contribution to the modeled probability of WHO-defined high-risk status (positive values indicate higher modeled probability and negative values indicate lower modeled probability), and color indicates the relative feature value from low to high.

In the explainable machine-learning workflow, candidate predictors were prespecified as BRI plus age, sex, and Model 3 covariates (education, marital status, alcohol use, diastolic blood pressure, heart rate, and triglycerides). We applied 10-fold cross-validated LASSO logistic regression using AUC as the optimization metric and selected the penalty parameter at λ1se to obtain a parsimonious subset of predictors. At λ1se, four predictors retained non-zero coefficients—diastolic blood pressure, age, BRI, and triglycerides (Figure 3A). The resulting random forest model achieved good discrimination (AUC = 0.784) (Figure 3B). SHAP analyses indicated that the selected predictors contributed differentially to model outputs; features were ordered by mean absolute SHAP value, yielding an interpretable importance ranking (diastolic blood pressure ranked 1 of 4 selected predictors, age ranked 2 of 4, BRI ranked 3 of 4, and triglycerides ranked 4 of 4) (Figure 3C). In the SHAP summary plot, each point represents one participant; the horizontal position indicates whether that feature shifts the modeled probability of WHO-defined high-risk status downward (negative SHAP value) or upward (positive SHAP value), whereas color indicates the relative feature value from low to high. This presentation provides both a global ranking of feature importance and an individual-level visualization of how each predictor contributes to model output.

## Discussion

In this cross-sectional analysis, a higher body roundness index (BRI) was consistently associated with greater odds of being classified as WHO-defined CVD high risk. We observed a non-linear dose–response pattern, an empirically derived operational cutoff BRI 4.151 (pragmatically grouped as ≥4), heterogeneity across cardiometabolic strata, and supportive evidence from an explainable machine-learning workflow.

### Principal findings

Our findings indicate that higher BRI was consistently associated with WHO-defined CVD high-risk status across multiple analytic approaches. The association persisted after stepwise adjustment, followed a non-linear pattern, and showed heterogeneity across selected cardiometabolic subgroups. From a discrimination perspective, BRI alone had limited standalone utility, whereas models incorporating additional routinely collected covariates performed better. The complementary machine-learning analyses further supported that BRI contributed relevant information within a broader multivariable framework.

### Interpretation and potential mechanism

BRI is derived from waist circumference and height and is intended to capture overall body shape and central adiposity (24–26). The observed non-linear pattern suggests that the association may become stronger once central adiposity reaches a higher level, which is biologically plausible because visceral fat accumulation, insulin resistance, adverse lipid metabolism, and hemodynamic burden may intensify disproportionately rather than linearly across the BRI distribution. A higher BRI likely reflects greater visceral fat accumulation and ectopic fat deposition, which are closely linked to insulin resistance, systemic low-grade inflammation, oxidative stress, dyslipidemia, and neurohormonal activation (24, 25, 27). These pathways can promote elevated blood pressure, impaired glucose metabolism, atherogenic lipid profiles, and endothelial dysfunction—features that cluster with higher predicted CVD risk in population screening. Importantly, BRI is not a direct physiological measure; rather, it may serve as an accessible anthropometric summary of adverse fat distribution that is feasible at scale in primary-care and community settings.

### Discrimination and operational threshold

The ROC analyses indicate that BRI has limited standalone utility for discriminating WHO-defined high-risk status, as the AUC of 0.590 is only modestly above chance. Accordingly, BRI should not be interpreted as a clinically sufficient stand-alone screening tool; rather, it may serve only as a low-cost adjunct within a broader multivariable risk assessment pathway. The Youden-index-derived cutoff of 4.151 should also be interpreted cautiously because it was data-driven and evaluated only within the same dataset, with internal bootstrap validation rather than external validation. We therefore regard this threshold as exploratory and useful primarily for descriptive grouping and secondary data analysis, not for immediate clinical adoption or treatment decision-making. Because the dose–response relationship was continuous and non-linear rather than stepwise, dichotomization should be viewed as a pragmatic analytic simplification rather than evidence of a biologically or clinically established threshold. Whenever feasible, retaining BRI as a continuous measure is likely to preserve information and statistical power.

The supplementary incremental analyses suggest that BRI may provide the greatest added value in simpler screening models, whereas its incremental contribution becomes modest once a more clinically informative base model is already available. The largely overlapping decision-curve patterns further indicate limited practical separation among BRI, BMI, and WC in terms of net benefit. In the sensitivity analysis excluding triglycerides and diastolic blood pressure, re-adding BRI still improved discrimination, supporting that its association was not entirely driven by overlap with these variables, although the overall clinical utility remained limited.

### Effect modification across subgroups

We observed stronger associations in men than in women, with significant interaction in both the continuous (*P* for interaction = 0.001) and dichotomized (*P* for interaction = 0.008) analyses (28). This pattern may reflect sex differences in fat distribution and metabolic susceptibility, as men tend to accumulate proportionally more visceral adiposity for a given waist-to-height profile (28–30). Interactions with diastolic blood pressure and triglycerides were also evident (both *P* for interaction <0.001), with attenuation of the BRI–high-risk association in the highest DBP and TG quartiles. One plausible interpretation is a saturation or “ceiling” effect: among individuals with marked hemodynamic or lipid abnormalities, the probability of being classified as high risk may already be elevated, reducing the incremental separation attributable to body shape. Alternatively, medication use, survivor bias, or measurement variability in extreme strata may contribute. Notably, interaction with heart rate was apparent when BRI was analyzed continuously (*P* for interaction = 0.020) but not after dichotomization, which is consistent with information loss when a continuous exposure is collapsed into a binary group. Overall, these subgroup patterns emphasize that BRI may behave differently across cardiometabolic phenotypes and warrant confirmation in prospective analyses.

The FDR-adjusted analyses further suggest that the most consistent heterogeneity signals were confined to sex and cardiometabolic strata defined by DBP and triglycerides, whereas more marginal subgroup differences were attenuated after correction. Accordingly, these subgroup findings should be interpreted as exploratory and hypothesis-generating rather than definitive, although the persistence of the sex, DBP, and TG interactions supports potentially meaningful variation in the BRI-high-risk association across metabolic and hemodynamic phenotypes.

### Explainable machine-learning insights

The explainable machine-learning analysis provides a complementary, classification-oriented perspective. Using the prespecified candidate predictors available in the screening dataset, LASSO selected a parsimonious subset that included diastolic blood pressure, age, triglycerides, and BRI. A random forest model trained on these selected predictors achieved good discrimination (AUC 0.784). SHAP analyses indicated that predictors contributed unequally to the predicted probability of being classified as WHO high risk: diastolic blood pressure and age showed the largest average contributions, while BRI remained a meaningful contributor. In terms of feature importance among the selected predictors, BRI ranked 3rd out of 4 by mean absolute SHAP value (i.e., a mid-pack contributor). These findings support the interpretation that BRI carries a relevant signal, while also underscoring that a risk-chart-based outcome is strongly related to hemodynamic and age-related features.

### Implications for community screening and primary care

Taken together, our findings support the potential use of BRI as an interpretable, low-cost anthropometric marker to support risk triage in large-scale community screening programs. In settings where staffing and laboratory capacity are constrained, calculating BRI from routine measurements could help prioritize individuals for more complete risk evaluation (e.g., formal risk-chart assessment, confirmatory blood testing when indicated, and targeted counseling). Because BRI alone has limited discrimination, its value is likely greatest as one component of a multivariable screening pathway rather than as an isolated decision rule. The observed sex heterogeneity also suggests that implementation strategies and decision thresholds may need to consider sex-specific risk patterns (31–33).

### Strengths and limitations

This study leverages a large, real-world screening cohort with standardized measurements and a pragmatic outcome definition aligned with public health practice. We assessed BRI using multiple complementary approaches—multivariable regression, restricted cubic splines, ROC-based operational thresholding with bootstrap uncertainty, subgroup/interaction analyses, and an explainable machine-learning workflow-providing convergent evidence for robustness and interpretability.

Several limitations merit consideration. First, the cross-sectional design precludes temporal inference, and the outcome is a risk-chart-based classification rather than adjudicated CVD events; therefore, the results should be interpreted as an association with high-risk status rather than evidence that BRI predicts future events. Second, the WHO risk-chart classification is partly driven by variables that correlate with adiposity (e.g., age and blood pressure). We intentionally avoided adjusting for all chart components to reduce circularity and to preserve a screening-oriented interpretation, but residual confounding by chart determinants may remain. Third, the operational cutoff was derived and evaluated within the same dataset and should therefore be considered exploratory. Because only internal validation was performed, this threshold should not be interpreted as ready for clinical adoption, and external validation is required before any broader implementation or decision-threshold use is considered. Finally, generalizability may be limited to a single-region community screening population in China with an age range of 35–75 years. Because body fat distribution and the anthropometric correlates of cardiometabolic risk may differ across ethnic groups and healthcare settings, the present findings should be extrapolated cautiously beyond similar Chinese community-based populations. External validation in other regions, ethnic groups, and clinical contexts is needed.

### Future directions

Future work should evaluate the incremental value of BRI beyond established risk tools using prospective follow-up and incident cardiovascular endpoints, including assessment of calibration, net reclassification, and clinical utility (e.g., decision-curve analyses). External validation in diverse populations is needed to determine whether the observed non-linear pattern, sex differences, and cardiometabolic interactions are reproducible. Implementation studies could further assess the feasibility, acceptability, and cost-effectiveness of incorporating BRI into community-based screening workflows, especially in resource-limited primary-care settings.

## Conclusion

In this large community-based screening population, higher BRI was positively associated with WHO-defined CVD high-risk status and displayed a non-linear risk gradient. Although BRI alone showed modest discriminatory performance, it may still have value as a low-cost adjunct marker when interpreted alongside other routinely collected indicators. An exploratory threshold may be useful for descriptive stratification or research-oriented triage, but it should not be considered ready for clinical adoption without external validation. Prospective studies with hard cardiovascular outcomes and external validation are warranted to confirm generalizability and to define the most effective implementation strategy.

## Data Availability

The data that support the findings of this study are available from the corresponding author Haoran Wang (d201278406@alumni.hust.edu.cn), upon reasonable request.

## Supplementary material

SUPPLEMENTARY FIGURE S1 Comparative receiver operating characteristic (ROC) and decision-curve analyses (DCA) of body roundness index (BRI), body mass index (BMI), and waist circumference (WC) for WHO-defined cardiovascular disease high-risk status. **(A)** ROC curves for anthropometric-only models. **(B)** ROC curves for Model 2 plus BRI, BMI, or WC, with the base model shown as a dashed grey line. **(C)** ROC curves for Model 3 plus BRI, BMI, or WC, together with the sensitivity model excluding triglycerides and diastolic blood pressure before adding BRI; the Model 3 base model is shown as a dashed grey line, and the sensitivity base model as a dashed brown line. **(D)** DCA for anthropometric-only models. **(E)** DCA for Model 2 plus BRI, BMI, or WC. **(F)** DCA for Model 3 plus BRI, BMI, or WC, together with the corresponding sensitivity model. Insets in panels A-C show the area under the curve (AUC) with 95% confidence intervals (CIs), change in AUC (ΔAUC), and continuous net reclassification improvement (NRI) relative to the panel-specific reference model. Model 2 included alcohol use and triglycerides; Model 3 additionally included marital status, education, diastolic blood pressure, and heart rate.

## Glossary

AUC: Area under the curve
BMI: Body mass index
Bpm: Beats per minute
BRI: Body roundness index
CI: Confidence interval
CVD: Cardiovascular disease
DBP: Diastolic blood pressure
HDL-C: High-density lipoprotein cholesterol
LDL-C: Low-density lipoprotein cholesterol
LASSO: Least absolute shrinkage and selection operator
Non-HDL-C: Non-high-density lipoprotein cholesterol
OR: Odds ratio
RCS: Restricted cubic spline
RF: Random forest
ROC: Receiver operating characteristic
SBP: Systolic blood pressure
SHAP: Shapely additive explanations
STROBE: Strengthening the Reporting of Observational Studies in Epidemiology
TC: Total cholesterol
TG: Triglycerides
WC: Waist circumference
WHO: World health organization
