# Supplementary figures and images for: Integrating epidemiologic modeling and explainable machine learning to evaluate body roundness index for WHO-defined high cardiovascular risk: evidence from the ChinaHEART-Luohe screening cohort
Source: Front Nutr. 2026 Apr 21;13:1818427. doi: 10.3389/fnut.2026.1818427 (PMC13139016; doi:10.3389/fnut.2026.1818427)

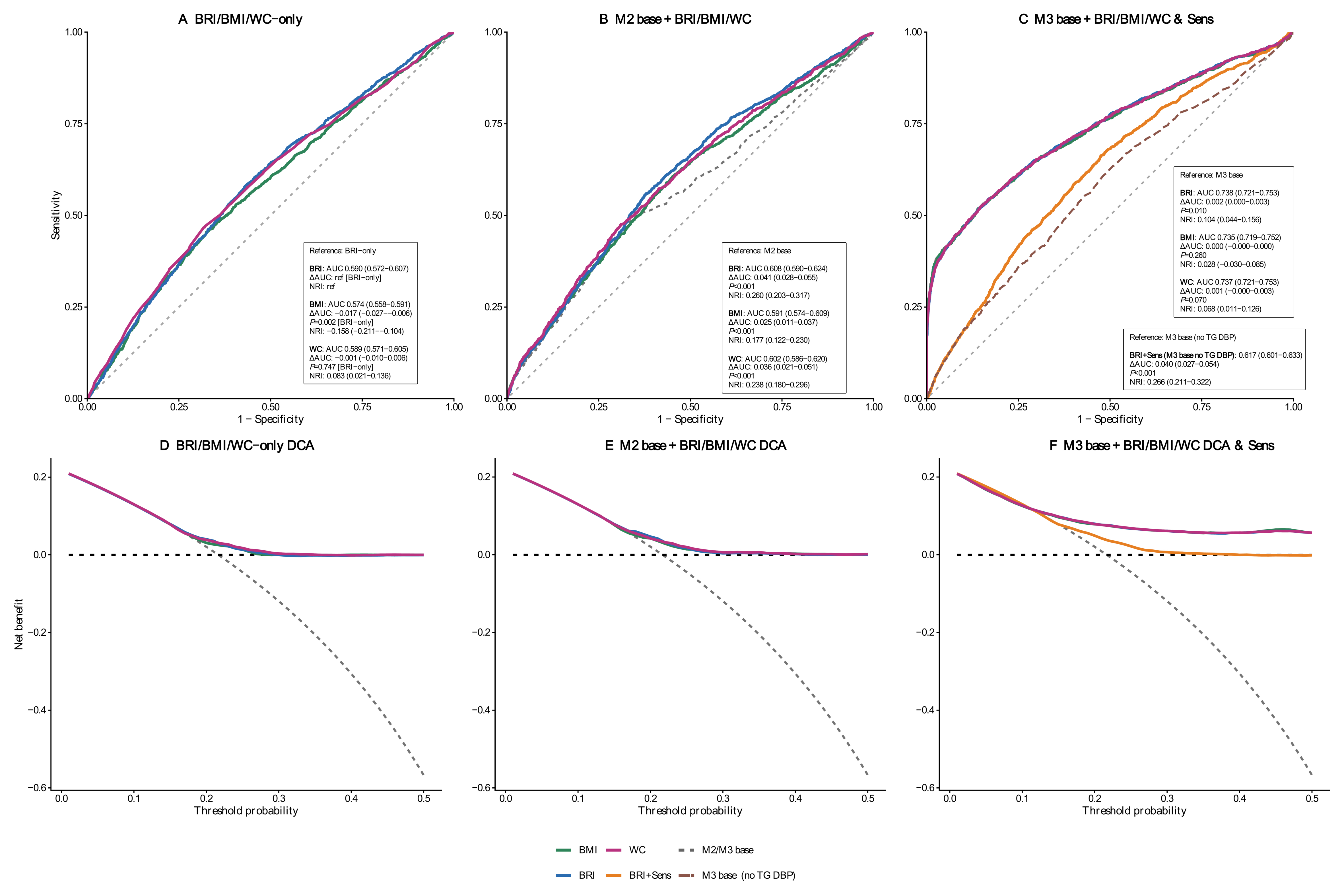

Supplement: SUPPLEMENTARY FIGURE S1 — Comparative receiver operating characteristic (ROC) and decision-curve analyses (DCA) of body roundness index (BRI), body mass index (BMI), and waist circumference (WC) for WHO-defined cardiovascular disease high-risk status. (A) ROC curves for anthropometric-only models. (B) ROC curves for Model 2 plus BRI, BMI, or WC, with the base model shown as a dashed grey line. (C) ROC curves for Model 3 plus BRI, BMI, or WC, together with the sensitivity model excluding triglycerides and diastolic blood pressure before adding BRI; the Model 3 base model is shown as a dashed grey line, and the sensitivity base model as a dashed brown line. (D) DCA for anthropometric-only models. (E) DCA for Model 2 plus BRI, BMI, or WC. (F) DCA for Model 3 plus BRI, BMI, or WC, together with the corresponding sensitivity model. Insets in panels A-C show the area under the curve (AUC) with 95% confidence intervals (CIs), change in AUC (ΔAUC), and continuous net reclassification improvement (NRI) relative to the panel-specific reference model. Model 2 included alcohol use and triglycerides; Model 3 additionally included marital status, education, diastolic blood pressure, and heart rate. [file Image_1.JPEG]
