# Supplementary material for: Integrating epidemiologic modeling and explainable machine learning to evaluate body roundness index for WHO-defined high cardiovascular risk: evidence from the ChinaHEART-Luohe screening cohort
Source: Front Nutr. 2026 Apr 21;13:1818427. doi: 10.3389/fnut.2026.1818427 (PMC13139016; doi:10.3389/fnut.2026.1818427)
Supplement: Supplementary file 2 [file Table_1.DOCX]

## *Supplementary Table S1: Subgroup analyses with FDR-adjusted P-values*

| **Analysis** | **Subgroup** | **Level** | **N** | **OR (95% CI)** | ***P* value** | **FDR-adjusted *P*** | ***P* for interaction** | **FDR-adjusted interaction *P*** |
| --- | --- | --- | --- | --- | --- | --- | --- | --- |
| BRI per 1-unit increase | Overall |  | 6,858 | 1.255 (1.201–1.312) | <0.001 |  |  |  |
|  | Age (quartiles) | Q1 (35.00–51.00) | 1,853 | 1.357 (1.219–1.509) | <0.001 | <0.001 | 0.108 | 0.124 |
|  |  | Q2 (51.00–58.00) | 1,598 | 1.180 (1.074–1.295) | <0.001 | <0.001 |  |  |
|  |  | Q3 (58.00–66.00) | 1,706 | 1.160 (1.065–1.262) | <0.001 | <0.001 |  |  |
|  |  | Q4 (66.00–76.00) | 1,701 | 1.256 (1.159–1.361) | <0.001 | <0.001 |  |  |
|  | Sex | Female | 4,272 | 1.212 (1.147–1.281) | <0.001 | <0.001 | 0.001 | 0.003 |
|  |  | Male | 2,586 | 1.423 (1.313–1.541) | <0.001 | <0.001 |  |  |
|  | Education | No | 1,292 | 1.171 (1.074–1.277) | <0.001 | <0.001 | 0.092 | 0.122 |
|  |  | Yes | 5,566 | 1.278 (1.212–1.347) | <0.001 | <0.001 |  |  |
|  | Married | No | 771 | 1.135 (1.004–1.283) | 0.042 | 0.049 | 0.086 | 0.122 |
|  |  | Yes | 6,087 | 1.274 (1.214–1.336) | <0.001 | <0.001 |  |  |
|  | Alcohol use | No | 6,478 | 1.247 (1.191–1.306) | <0.001 | <0.001 | 0.775 | 0.775 |
|  |  | Yes | 380 | 1.281 (1.072–1.529) | 0.006 | 0.008 |  |  |
|  | Diastolic BP (quartiles) | Q1 (43.50–76.50) | 1,813 | 1.366 (1.219–1.531) | <0.001 | <0.001 | <0.001 | <0.001 |
|  |  | Q2 (76.50–83.50) | 1,724 | 1.358 (1.211–1.524) | <0.001 | <0.001 |  |  |
|  |  | Q3 (83.50–91.00) | 1,676 | 1.081 (0.977–1.196) | 0.134 | 0.146 |  |  |
|  |  | Q4 (91.00–141.00) | 1,645 | 0.959 (0.891–1.032) | 0.265 | 0.277 |  |  |
|  | Heart rate (quartiles) | Q1 (42.00–70.00) | 1,815 | 1.276 (1.164–1.399) | <0.001 | <0.001 | 0.020 | 0.040 |
|  |  | Q2 (70.00–75.50) | 1,754 | 1.345 (1.222–1.481) | <0.001 | <0.001 |  |  |
|  |  | Q3 (75.50–82.50) | 1,633 | 1.306 (1.194–1.429) | <0.001 | <0.001 |  |  |
|  |  | Q4 (82.50–119.00) | 1,651 | 1.125 (1.037–1.221) | 0.005 | 0.006 |  |  |
|  | Triglycerides (quartiles) | Q1 (0.50–1.14) | 1,730 | 1.251 (1.129–1.385) | <0.001 | <0.001 | <0.001 | <0.001 |
|  |  | Q2 (1.14–1.51) | 1,674 | 1.235 (1.117–1.366) | <0.001 | <0.001 |  |  |
|  |  | Q3 (1.51–2.03) | 1,714 | 1.470 (1.336–1.616) | <0.001 | <0.001 |  |  |
|  |  | Q4 (2.03–5.65) | 1,683 | 1.042 (0.962–1.128) | 0.314 | 0.314 |  |  |
| BRI ≥4 vs <4 | Overall |  | 6,858 | 1.809 (1.607–2.035) | <0.001 |  |  |  |
|  | Age (quartiles) | Q1 (35.00–51.00) | 1,853 | 2.284 (1.755–2.972) | <0.001 | <0.001 | 0.051 | 0.101 |
|  |  | Q2 (51.00–58.00) | 1,598 | 1.497 (1.181–1.898) | <0.001 | 0.001 |  |  |
|  |  | Q3 (58.00–66.00) | 1,706 | 1.468 (1.163–1.855) | 0.001 | 0.002 |  |  |
|  |  | Q4 (66.00–76.00) | 1,701 | 1.818 (1.455–2.271) | <0.001 | <0.001 |  |  |
|  | Sex | Female | 4,272 | 1.610 (1.379–1.879) | <0.001 | <0.001 | 0.009 | 0.023 |
|  |  | Male | 2,586 | 2.220 (1.847–2.668) | <0.001 | <0.001 |  |  |
|  | Education | No | 1,292 | 1.484 (1.135–1.940) | 0.004 | 0.005 | 0.133 | 0.178 |
|  |  | Yes | 5,566 | 1.868 (1.637–2.131) | <0.001 | <0.001 |  |  |
|  | Married | No | 771 | 1.472 (1.040–2.082) | 0.029 | 0.033 | 0.223 | 0.255 |
|  |  | Yes | 6,087 | 1.854 (1.635–2.102) | <0.001 | <0.001 |  |  |
|  | Alcohol use | No | 6,478 | 1.767 (1.562–1.998) | <0.001 | <0.001 | 0.630 | 0.630 |
|  |  | Yes | 380 | 1.978 (1.269–3.084) | 0.003 | 0.003 |  |  |
|  | Diastolic BP (quartiles) | Q1 (43.50–76.50) | 1,813 | 2.195 (1.642–2.932) | <0.001 | <0.001 | <0.001 | <0.001 |
|  |  | Q2 (76.50–83.50) | 1,724 | 1.897 (1.414–2.543) | <0.001 | <0.001 |  |  |
|  |  | Q3 (83.50–91.00) | 1,676 | 1.196 (0.918–1.557) | 0.184 | 0.201 |  |  |
|  |  | Q4 (91.00–141.00) | 1,645 | 0.956 (0.777–1.175) | 0.667 | 0.667 |  |  |
|  | Heart rate (quartiles) | Q1 (42.00–70.00) | 1,815 | 1.794 (1.418–2.270) | <0.001 | <0.001 | 0.130 | 0.178 |
|  |  | Q2 (70.00–75.50) | 1,754 | 2.055 (1.589–2.659) | <0.001 | <0.001 |  |  |
|  |  | Q3 (75.50–82.50) | 1,633 | 2.012 (1.582–2.559) | <0.001 | <0.001 |  |  |
|  |  | Q4 (82.50–119.00) | 1,651 | 1.443 (1.157–1.800) | 0.001 | 0.002 |  |  |
|  | Triglycerides (quartiles) | Q1 (0.50–1.14) | 1,730 | 1.635 (1.289–2.073) | <0.001 | <0.001 | <0.001 | <0.001 |
|  |  | Q2 (1.14–1.51) | 1,674 | 1.647 (1.285–2.109) | <0.001 | <0.001 |  |  |
|  |  | Q3 (1.51–2.03) | 1,714 | 3.046 (2.333–3.977) | <0.001 | <0.001 |  |  |
|  |  | Q4 (2.03–5.65) | 1,683 | 1.069 (0.850–1.343) | 0.568 | 0.593 |  |  |

Supplementary Table S1. Unadjusted subgroup analyses of BRI and WHO-defined CVD high-risk status with BH-FDR adjusted *P* values. FDR adjusts *P* values rather than OR estimates.
